# Identifying Interaction Clusters for MiRNA and MRNA Pairs in TCGA Network

**DOI:** 10.3390/genes10090702

**Published:** 2019-09-11

**Authors:** Xinqing Dai, Lizhong Ding, Hannah Liu, Zesheng Xu, Hui Jiang, Samuel K Handelman, Yongsheng Bai

**Affiliations:** 1 Department of Mathematics and Computer Science, Indiana State University, Terre Haute, IN 47809, USA; 2 Department of Biology, Indiana State University, Terre Haute, IN 47809, USA; 3 Carmel High School, 520 E. Main St. Carmel, IN 46032, USA; 4 Seven Lakes High School, 9251 S Fry Rd, Katy, TX 77494, USA; 5 Department of Biostatistics, University of Michigan, Ann Arbor, MI 48109, USA; 6 Department of Internal Medicine, University of Michigan, Ann Arbor, MI 48109, USA

**Keywords:** miRNA, mRNA, liver hepatocellular carcinoma, gene regulation, clustering algorithm, The Cancer Genome Atlas

## Abstract

Existing methods often fail to recognize the conversions for the biological roles of the pairs of genes and microRNAs (miRNAs) between the tumor and normal samples. We have developed a novel cluster scoring method to identify messenger RNA (mRNA) and miRNA interaction pairs and clusters while considering tumor and normal samples jointly. Our method has identified 54 significant clusters for 15 cancer types selected from The Cancer Genome Atlas project. We also determined the shared clusters across tumor types and/or subtypes. In addition, we compared gene and miRNA overlap between lists identified in our liver hepatocellular carcinoma (LIHC) study and regulatory relationships reported from human and rat nonalcoholic fatty liver disease studies (NAFLD). Finally, we analyzed biological functions for the single significant cluster in LIHC and uncovered a significantly enriched pathway (phospholipase D signaling pathway) with six genes represented in the cluster, symbols: *DGKQ*, *LPAR2*, *PDGFRB*, *PIK3R3*, *PTGFR* and *RAPGEF3*.

## 1. Introduction

Transcription from gene to messenger RNA (mRNA) and translation from mRNA to protein are two essential stages for cells to perform biological functions. Non-coding RNAs (e.g., microRNA (miRNA), long non-coding RNA (lncRNA)) are often involved in many cellular processes, but mainly in post-transcriptional regulation. MiRNAs, which are short (17–22 nt) highly processed oligonucleotides, play their regulatory roles through either degradation or inhibition of protein translation of the targeting mRNAs [1].

The messenger RNA (mRNA), an outcome of gene transcription, is essential to performing biochemical functions in the cell. Different regulatory RNAs (e.g., miRNA and lncRNA) are important driving factors for the stable and successful translation of mRNA in a cell. Therefore, regulatory RNAs play a vital role in mRNA activation and suppression. Among these regulatory RNAs, the miRNA class is intensively studied at both the sequence and functional level. These MiRNAs are involved in post-transcriptional regulation of the target mRNA by two known mechanisms; the degradation of target mRNA and suppression of protein translation [1]. Thus, regulation of the miRNA and mRNA network is complex. A single miRNA can target many mRNAs, while many miRNAs are able to cooperatively target a single mRNA, in both degradation and inhibition contexts. This allows for fine-tuned gene expression regulation [2]. Understanding of these mechanisms has advanced significantly with the advent of high-throughput microarray-based technologies such as expression profiling.

**Figure 1:**
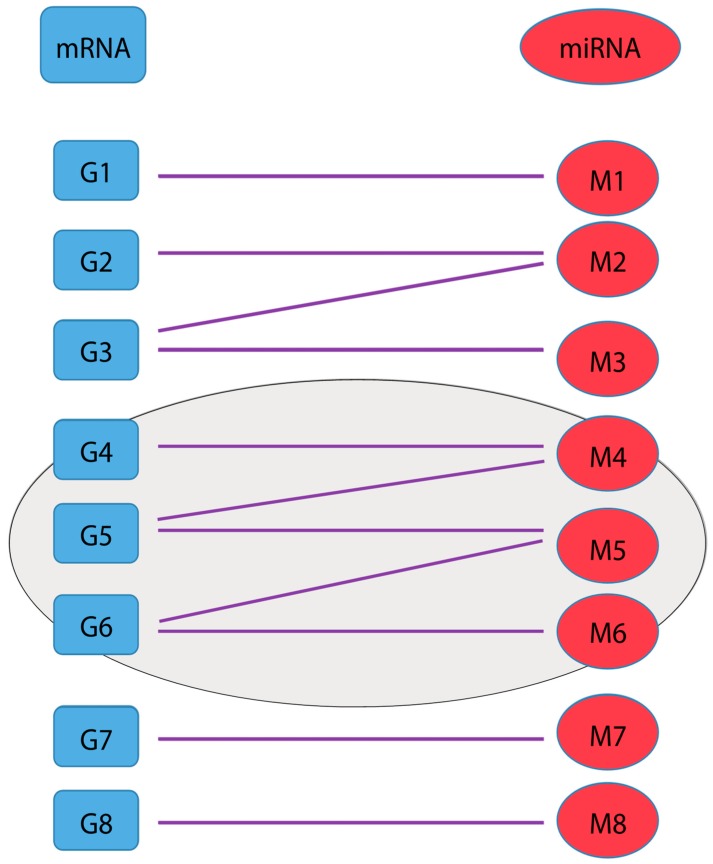
Bipartite diagram for messenger RNA (mRNA) (blue, left) and microRNA (miRNA) (red, right) interaction pairs, with a cluster identified by a purple ellipse.

Clusters in an mRNA–miRNA interaction network are often interaction complexes and/or parts of pathways. If interaction pair cluster(s) are significantly rendered in both tumor and normal samples, they could be important in the context of biological processes and/or cancers. Therefore, identifying such significant interaction clusters will identify genes and miRNAs functionally associated to various cancer molecular subtypes, with diagnostic and therapeutic implications. An undirected graph can be used to represent gene and miRNA relationships in an interaction network. Specifically, interactions between genes and miRNAs are rendered as a bipartite graph with genes or miRNAs as vertices and their interactions as edges (Figure 1). Thus, each vertex representing a gene or miRNA is connected to at least one of each other node since one gene or miRNA has at least one interaction with its interaction partner. Clusters in a network are formed by sets of vertices and edges with interconnections.

Clustering techniques are widely used to provide a reasonable understanding about gene function, regulation, and cellular process. A group of similar objects makes an exclusive class of objects (similar genes, same cluster), whereas dissimilar objects are distributed into different clusters. The analysis of mRNA–miRNA interaction is complex. Most existing graph-based clustering algorithms (also called graph partition algorithms or community detection algorithms) consider the topology of only a single instance (e.g., gene or miRNA) and treat all of nodes equivalently in the graph. Moreover, the complexity of the change of the correlation coefficients and expression values of the mRNA–miRNA pairs between tumor and normal samples is still not resolved, and this hinders the potential clinical applications. There is an urgent need to develop innovative methodologies and tools to accurately cluster mRNA–miRNA interaction pairs into functional miRNA–mRNA regulatory modules while analyzing tumor and normal samples jointly.

Previous studies on clustering such data were mainly based on microarray gene expression data [3,4] and can just analyze only samples within one category (e.g., tumor samples alone). In this study, a novel concurrent simultaneous clustering (or co-biclustering) method for identifying gene and miRNA interaction clusters in a network was designed and implemented. This novel method considers tumor and normal samples jointly in the same network.

## 2. Materials and Methods

### 2.1. Significant mRNA–miRNA Pairs Selection for Input Data

The Cancer Genome Atlas (TCGA) (http://cancergenome.nih.gov) datasets were employed to generate the miRNA and mRNA expression files for evaluation. The TCGA data level 3 was used for the expression results. The University of North Carolina at Chapel Hill (UNC) and the Baylor College Human Genome Sequencing (BCGSC) data were considered for RNA-Seq and miRNA-Seq data respectively. Out of 33 cancer types (Supplementary file 1), we obtained RNA-Seq and miRNA-Seq data for 15 cancer types (eight of these cancer types were previously characterized [5], and seven additional cancer types are analyzed using the novel methods in this report) used for downstream analysis, after excluding cancer types which do not have corresponding tumor or normal samples. A computer C program was developed to calculate the Pearson correlation coefficient (CC). The targets prediction outcomes were testified using Targetprofiler [6], TargetScan [7] and miRanda [8]. We employed the same approach used in our previous study to claim target relationships if there is the match between pre-miRNA from TCGA datasets and the same or closely related mature miRNA from target prediction databases. The same target prediction criterion was applied if the prediction was supported by at least one of three databases mentioned above [5]. To filter out miRNA and mRNA pairs with insignificant CC, an R Script was written to compute the statistical significance (*p*-values and *Q*-values or false discovery rates (FDR)) for each calculated CC and select statistically significant pairs of miRNA and mRNA.

To check the expression change (up-regulation, down-regulation, and no change) of transcripts in cancer(s), we calculated average expression or fold change (FC) values for miRNAs and mRNAs in tumor and normal samples. The significant (FDR < 0.1) miRNA and mRNA pairs with inverse correlations between tumor and normal samples and with FC values greater than 1.5 were selected as input for the clustering algorithm to process.

### 2.2. Cluster Identification and Scoring Algorithm

We modified the Louvain algorithm [9] employed by NetworkX (https://networkx.github.io/) by considering the correlation coefficient values for both tumor and normal samples simultaneously to detect “communities” or clusters upon processing significant pairs selected for each of the 15 cancers in TCGA project. We used the following customized scoring algorithm to calculate the “score” for each detected cluster. Our assumption is that clusters consisting of gene and miRNA pairs having the most fold changes in their expression and with the highest correlations could be associated with cancers. Specifically, the scoring algorithm will (1) Calculate expression and CC values for genes and miRNAs in both tumor and normal samples; (2) Calculate and normalize deviation scores for features (node and edge) in clusters; (3) Compute the total scores for each classified cluster in the best partition; (4) Calculate the statistical significance for classified clusters and report top scored and significant clusters in the best partition.

$$score=\overset{m}{\underset{i=1}{\sum }}\left|\frac{\ln\left(mRNA\_FC_{i}\right)\;}{\sqrt{1+\ln(mRNA\_FC_{i}{}^{2})\;}}\right|+\overset{n}{\underset{i=1}{\sum }}\left|\frac{\ln\left(miRNA\_FC_{i}\right)\;}{\sqrt{1+\ln(miRNA\_FC_{i}{}^{2})}}\right|+\overset{q}{\underset{i=1}{\sum }}(\left|T\_CC_{i}\right|+\left|N\_CC_{i}\right|)$$

where *mRNA_FC_i_* is the fold change of gene expression, *miRNA_FC_i_* is the fold change of miRNA expression, *T_CC_i_* is the CC value in tumor samples, and *N_CCi* is the CC value in normal samples. *m* is the number of genes, *n* is the number of miRNAs, and *q* is the total number of edges in the bicluster.

There were four variables: mRNA fold change between normal and tumor samples (mRNA_FC), miRNA fold change between normal and tumor samples (miRNA_FC), mRNA and miRNA correlation coefficient in tumor sample (T_CC), and mRNA and miRNA correlation coefficient in normal sample (N_CC) which were used for score calculation. We added both correlation coefficient values (absolute values for negative correlation coefficient) into the formula to enhance the cluster score. We took absolute values of each variable, then we normalized mRNA_FC and miRNA_FC to make sure their values are between 0 to 1. In addition, we only selected the mRNA–miRNA pairs with large fold changes (cutoff value 1.5) for their miRNA and mRNA expression changed in the opposite direction ((FC > 1.5 for mRNA and FC < 1.5 for miRNA) OR (FC > 1.5 for miRNA and FC < 1.5 for mRNA)) to run the clustering algorithm. A high score was evidence that the observed cluster is associated with biologically-driven co-expression.

### 2.3. Statistical Analysis

**Figure 2:**
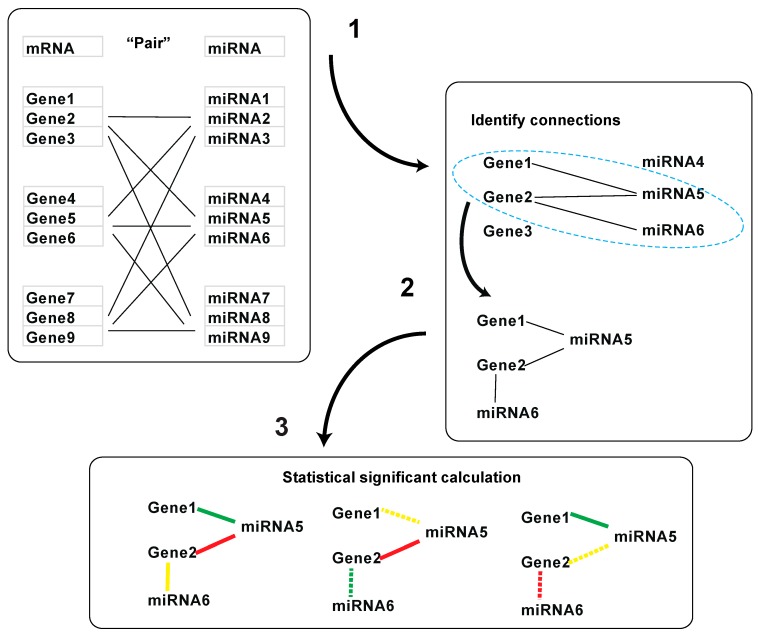
Workflow for statistical significance test for gene and miRNA interaction clusters: (1) miRNA and mRNA pairs with target relationship; (2) cluster identification; (3) permutation test.

The significance of a detected cluster was calculated using a permutation test approach. Specifically, the *p*-value P_k_ for a cluster C_k_ is defined as the probability of observing a cluster with a score of at least S_k_, if the graph is generated “randomly” (i.e., when we do not expect to see any cluster in it). We used the permutation test to assess the statistical significance of highly scored clusters by sampling enough randomized graphs which will be generated by shuffling (or permuting) the nodes (mRNAs and miRNAs) without changing the topology of the graph. We then ran our partition and scoring algorithm on each of the randomized graphs. The *p*-value for a cluster detected by our algorithm in the original graph is estimated as the proportion of randomized graphs that has a cluster whose score is equal to or larger than the one detected in the original graph (Figure 2). For example, if we generated 1,000,000 “random” graphs, and among them there are 1000 “random” graphs from which we got a cluster with a score of at least S_k_, then the *p*-value for a cluster with a score of S_k_ in the original (i.e., “non-random”) graph is P_k_ = 1001/1,000,001 = 0.001. Benjamini-Hochberg procedure was used to calculate false discovery rate (FDR) for adjusting the *p* values. For a low *p*-value, we reject the null hypothesis that no biological clusters are reflected in the observed data.

### 2.4. Determination of Shared Clusters Across Tumor Types and/or Subtypes

It was also our goal to identify clusters with common genes and miRNAs across several cancer types since they could be associated with several cancer diseases. To compare two graphs or clusters it was necessary to identify corresponding genes and miRNAs across two different clusters. A list of correspondence between the genes and miRNAs and their interactions can be regarded as a set of edges that connect the vertices across two different clusters. 

**Figure 3:**
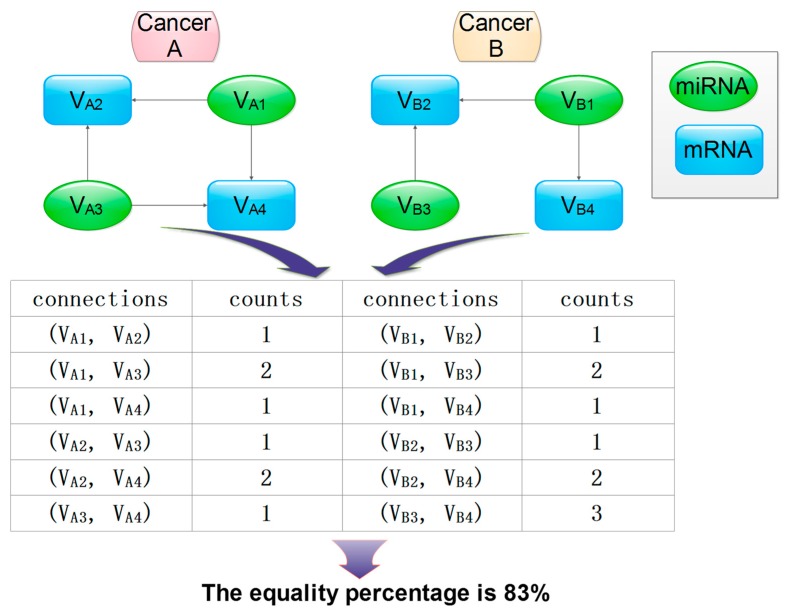
A schematic representation of the graph comparison algorithm to detect correlated clusters or local similarities in two graphs. The count was calculated as the number of connections between two nodes.

Figure 3 shows the workflow of our proposed graph comparison method. Specifically, the steps of graph comparison algorithm will be implemented as follows:

1. Identify corresponding vertices (genes and miRNAs) and edges (their interactions) that connect their vertices in clusters between two different cancers (A and B);
2. Construct matrices to store vertices and edges;
3. Calculate the shortest “distance” as the number of edges between any two vertices for each cluster in cancer A and B, respectively;
4. Determine whether two clusters match based on their matching percentage (defined as the ratio of the number of corresponding vertex pairs with equal distance out of total matched vertex pairs).

The strategy has been expanded to compare clusters between multi-cancers. Specifically, we constructed a matrix with rows represented as connections in the common cluster across cancers and columns as cancer types. The cell in the matrix was filled with “distance” values. The number of common values in one row over the total number values of that row was computed as the row (r) percentage across multiple cancers; the total (t) percentage of the matrix was the ratio of sums of the numerator of all r percentages meeting the user-defined cutoff criterion over the total number of rows in the matrix.

### 2.5. Check the Overlap Between miRNAs Reported in LIHC and Differentially Expressed miRNAs from Studies of Human and Rat with Nonalcoholic Fatty Liver Disease

Nonalcoholic fatty liver disease (NAFLD) can lead to liver inflammation resulting in fibrosis, cirrhosis and finally in hepatocellular carcinoma [10,11]. Across a range of expression studies, pathways implicated in this progression include circadian rhythms [12], oncogenes and toll-like receptors [13], immune activation more generally [14], and reorganization of the extracellular matrix [15]. This range of associations shows that the molecular etiology of this progression is still a matter of controversy; but it is credible that molecular processes associated with early stages in this disease progression contribute directly to the manifestation of the associated cancer. Therefore, we took the significant clusters reported in TCGA LIHC datasets and searched for published miRNA lists from human associated with NAFLD [16] and Type 2 diabetes (T2D) [17], and other liver diseases [18].

In addition, we also checked the conservation of miRNA reported in the significant cluster of LIHC. In a study about the liver of Wistar rats [19], authors identified lists of most abundant miRNAs differentially expressed in NAFLD and normal rat liver and miRNAs with the largest F-change between NAFLD and normal liver. We used the above list to make the comparison. 

## 3. Results

### 3.1. Inversely Correlated miRNA and mRNA Pairs with Opposite Fold Change

We ran customized correlation calculation and database prediction scripts to generate the filtered miRNA and mRNA co-expression data for the 15 selected cancer types. At an FDR of 0.1, we found 92,751 inversely-correlated miRNA and mRNA pairs. Of these, 45,882 pairs also showed opposing fold-change between tumor and control samples (Table 1 and Supplementary file 2).

### 3.2. Cluster Detection Results

We initially generated the miRNA–mRNA pairs using the approach described in our previous paper [5]. Specifically, we selected the significant miRNA–mRNA pairs with their expression correlation in tumor and normal samples that were inverse and the fold of change of expression of both mRNA and miRNA were great than 1.5 for cluster identification. Upon running the community detection Louvain algorithm [9] with our defined cluster score, the detected “communities” or clusters upon processing significant pairs selected in each of the 15 cancers in TCGA project are shown in Table 2.

For LIHC, the algorithm detected the largest number (114) of clusters but only one significant (*q*-value < 0.1) cluster: LIHC_57. LUAD and BRCA had the largest cluster sizes (908 for LUAD and 628 for BRCA). COAD contained the smallest number (20) of clusters. LUAD also has the highest number (9) of significant clusters represented the significant ratio 43% (9/21) of initial clusters (Table 2).

**Figure 4:**
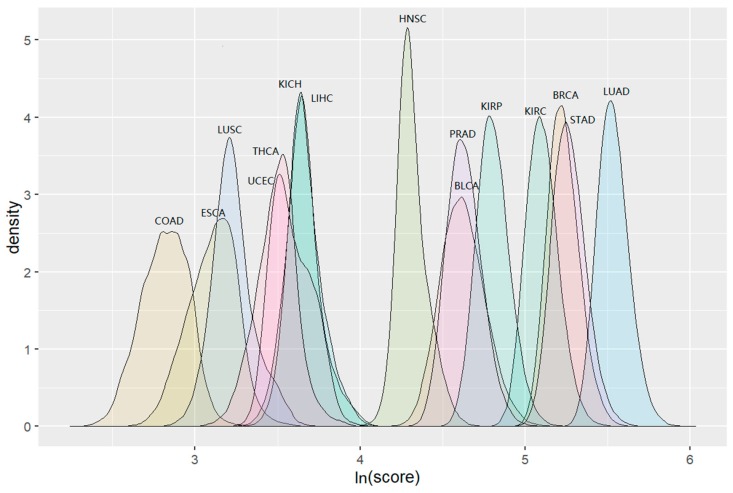
The distribution of cluster scores with max score values when running Louvain algorithm 10,000 times for 15 cancers.

The COAD clusters had the lowest score distribution and the scores for clusters in LUAD were shown to be the highest. This pattern was consistent with the distribution trend of the numbers of their clusters. The distribution of “maximum” scores for detected clusters during permutation tests in 15 cancers is shown in Figure 4. The detailed information including cluster sizes and cluster scores for identified clusters is shown in Supplementary file 3.

### 3.3. Cross-Cancer Comparison Results

**Figure 5:**
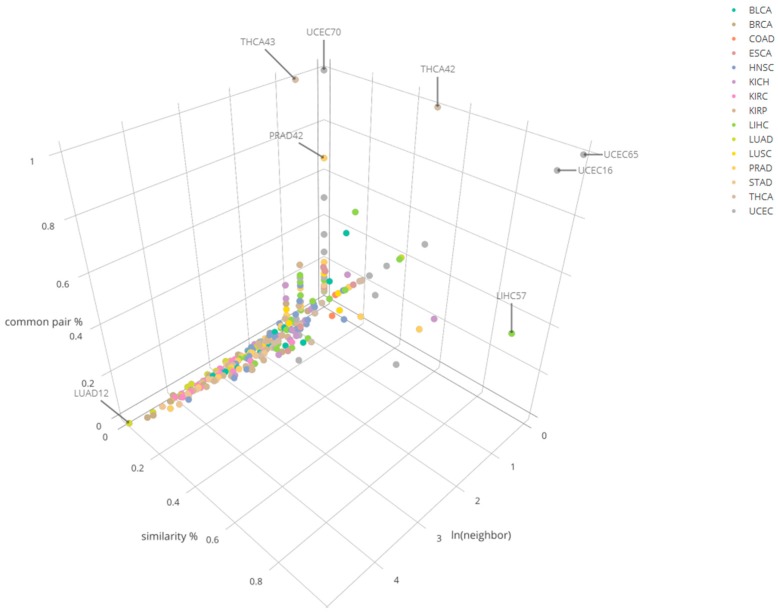
Cluster comparison results of 15 cancers for three different categories in the number of common mRNA–miRNA pairs across the comparison.

Under the cutoff criteria of the total percentage 0.5 and row percentage 0.6, we have identified 393 clusters which have their pairs available in at least one of the other cancers for comparison. From LUAD_12 cluster had the largest number of matchable clusters or neighbors (152) with at least one common pair identified. LIHC_57 showed the highest similarity percentage *vs* having high total common percentage among the cluster pool with matchable clusters 2 or less (Figure 5). In LIHC_57, LUAD_12 was identified to be one of similar clusters with it. Specifically, *PCSK1N* and *hsa-mir-378* pair is reported in common between LIHC_57 and LUAD_12. *GPR143* and *hsa-mir-378* pair was also in common between LIHC_57, KIRC_33, and KIRP_49, which indicate these cancer clusters could have common driving transcription regulation patterns due to the fact that they share common miRNA–mRNA pairs. The detailed information across multiple cancers is reported in Supplementary files 4–18. A list of compared clusters along with three metrices (number_of_neighbors, similarity_percentage, common_pair_percentage) is reported in Supplementary file 19.

### 3.4. Investigation of miRNAs and Their Targets Overlap Between Lists in LIHC and the Ones Reported from the Study in Human with Nonalcoholic Fatty Liver Disease 

Given that LIHC has the largest number (114) of clusters, we are interested in checking if any miRNAs/genes reported in LIHC are also prevalent in other liver associated diseases such as, NAFLD. We compared our miRNAs (224) in LIHC with the list of hepatic miRNAs (44) upregulated in human with NAFLD [16], and total 11 out of 41 (3 of them (2 Epstein–Barr viruses: ebv-miRBART18-3p and ebv-miRBART17-3p and 1 Herpes simplex virus: hsv2-miR-H20 were excluded in the analysis) miRNAs or 32 target pairs were identified to be present in LIHC clusters (Table 3). In checking the miRNA list (*hsa-mir-17*, *hsa-mir-20a*, *hsa-mir-20b*, *and hsa-mir-122*) upregulated in type 2 diabetes mellitus (T2DM) patients with NAFLD complication reported in another study [17], all these miRNAs have been identified in our LIHC cluster list. In addition, a well-known miRNA (*hsa-mir-22*) involved in hepatocellular carcinoma a cell migration and invasion [18] in liver disease has been identified in our LIHC cluster list. 

### 3.5. Investigation of miRNA Overlap Between Differentially Expressed miRNA List in Rat with Nonalcoholic Fatty Liver Disease and the Ones in LIHC 

We also checked the results for miRNA–mRNA in LIHC against the miRNAs (21) identified in the study [19] to see if there are any miRNAs associated with liver cancer which are also associated with NAFLD in rat.

Upon checking the gene/miRNA list reported in a study for rat with NAFLD, out of the 10 most abundant miRNAs differentially expressed in NAFLD and normal rat liver provided in the above mentioned study [19], six miRNAs (*mir-122*, *let-7c*, *let-7b*, *mir-192*, *mir-29a*, *mir-21*) were also identified to be present in LIHC clusters; out of the 10 miRNAs with the largest F-change between NAFLD and normal liver, four miRNAs (*mir-132*, *mir-99a*, *mir-200c*, *mir-145*) were also reported to be present in LIHC clusters. A list of overlapping miRNAs and their targeted gene pairs is reported in Table 4.

### 3.6. Cluster Functional Analysis for LIHC

We performed functional annotation and pathway analysis for 56 genes reported in cluster LIHC_17 using ClusterProfiler [20]. From the annotation result, four genes (*PDGFRB*, *PIK3IP1*, *PIK3R3*, *WDR91*) tagged with the Gene Ontology (GO) term biological process “regulation of phosphatidylinositol 3-kinase activity” and “lipid kinase activity” (*q*-value < 0.01) and “regulation of phospholipid metabolic process” (*q*-value = 0.02) were significantly enriched. In addition, three genes (*ACVRL1*, *COL3A1*, *PDGFRB*) have been reported to be significantly enriched (*q*-value = 0.04) with GO term “aorta morphogenesis”. In searching for pathway enrichment for this gene list, a significant (*q*-value < 0.002) pathway “phospholipase D signaling pathway” (KEGG ID: hsa04072) has been identified to be enriched for six genes (*DGKQ*, *LPAR2*, *PDGFRB*, *PIK3R3*, *PTGFR*, *RAPGEF3*). The pathways downstream of phospholipase D (PLD) are involved in oncogenic transformation. The research showed that membrane-associated phospholipase D can be activated by the small Guanosine-5’-triphosphate (GTP)-binding protein RhoA in rat liver [21]. *PDGFRB* or platelet-derived growth factor receptor B is a protein-coding gene and essential for normal development of the cardiovascular system. In a recent study [22], it has been reported to have the function of stimulating invasion and liver metastasis formation of mesenchymal-like colorectal tumor cells in mice. The gene *PIK3R3* has been reported to play an important role in colorectal cancer metastasis [23]. Blocking *PIK3R3* can prevent colorectal cancer liver metastasis in animal model [24]. A list of miRNAs targeting six genes involved in Phospholipase D signaling pathway is reported in Table 5.

## 4. Discussion

Existing tools cannot reveal the biological roles (e.g., cancer association) of pairs of genes and miRNAs due to their lack of consideration of the “inverse/altered regulation” between tumor and normal samples concurrently. Our proposed method considers the topology of genes in the network and takes as input inversely regulated target pairs containing their target prediction relationship predicted by several target database prediction algorithms to identify significant target pairs and elucidate cancer and disease associated signatures of clusters.

As a direct clinical application on our novel method, we have conducted the functional annotation for the significant clusters identified in BRCA datasets. Our analysis has confirmed that breast cancer related GO terms (cell cycle and chromosome) are enriched in some of the identified significant clusters. These clusters often contain more previously reported breast cancer risk genes than other identified significant clusters not enriched with breast cancer GO terms.

As biomarkers (of cancer, of cancer progression, of cancer resistance to chemotherapy, etc.), clusters of correlated expression levels will generically be more robust than any individual marker [25]. In conventional blood biochemical biomarkers, well-studied examples where ratios of related markers outperform individual markers include such liver disease scores as APRI [26] and FIB-4 [27], each of which includes the ratio of aspartate aminotransferase (AST) to platelets in the blood. As the field matures, the methods developed here will support similar and more robust ratio biomarkers as a natural extension of coding-gene differential expression [28].

There are previous studies of miRNA and mRNA regulatory networks in cancer. Those studies generally use expression profiles of miRNA and mRNAs and different clustering algorithms and/or statistical analyses to identify the potential miRNA–mRNA modules or clusters. Such studies usually also perform miRNA–mRNA pair selection based on miRNA–mRNA interaction experimental databases or prediction algorithms, functional enrichment analyses of the genes or proteins, disease association, and other analyses in order to relate the miRNA and mRNAs in modules to the cancer types/subtypes of interest or survival probability. Specifically, in a study of colorectal cancer, the rough hypercuboid based supervised clustering algorithm (RH-SAC) was used to generate clusters of functionally similar miRNAs or mRNAs whose coherent expression can further efficiently classify the samples [29]. In a study of multiple myelomas, through integrative analysis of GO biological processes, miRNA–mRNA targeting relationship, and matched miRNA and mRNA expression data, the ping-pong algorithm and multiobjective genetic algorithm were integrated to detect subtype-specific miRNA–mRNA regulatory modules [30]. In a study of glioblastomas, mRNA, miRNA, and protein expression profiles were integrated to identify regulatory networks. Cancer-related miRNAs were ranked based on the amount of identified correlated genes, including both protein and mRNA datasets. Then modules containing mRNAs, proteins, and miRNAs, in which the three molecular types are highly correlated, were clustered by the SAMBA bi-clustering algorithm, a Bayesian network model, and an extended step in which proteins are included into mRNA sample modules prior to the miRNAs’ inclusion [31]. Compared to other studies, our research is unique and novel in terms of selection of the significant miRNA–mRNA pairs with their expression correlation in tumor and normal samples that were inverse and the fold of change of expression of both mRNA and miRNA were great than 1.5. We used the modified Louvain algorithm to detect “communities” or clusters cluster from the bipartite graph of miRNA and mRNA vertices based on their correlation coefficient values for both tumor and normal samples simultaneously. We scored the detected clusters to see if they are significant. A graph comparison algorithm in our study was expanded to compare clusters across cancer types.

## 5. Conclusions

We believe our study is the first attempt to employ the idea of “inverse/altered regulation” and integrate multiple cancer specific databases with mRNA–miRNA interaction. Next, we plan to develop a powerful and user-friendly mRNA–miRNA functional annotation tool for visualizing cluster interactions in both tumor and normal samples of various cancer types. This effort will not only give a detailed presentation of miRNA associated with various types of human cancers, but will also provide a comprehensive analysis of gene ontology and mRNA-cancer associations.

By providing comprehensive and accessible tools to confront this growing class of cancer sequencing big data, our results provided a list of candidate cancer-associated genes and miRNAs with their biological functions and could shift current research and/or clinical practice paradigms. Our proposed method is applicable across a range of diseases and cancers and has uniquely distinctive advantages over existing tools. This will likewise contribute to new bioinformatics methodologies for identifying cancer driver genes in personal genomes in which clinicians seek to develop better treatment strategies.

## Figures and Tables

**Table 1:** The statistics for number of miRNA–mRNA pairs in 15 selected cancers from The Cancer Genome Atlas (TCGA).

Cancer Types	Number of miRNA–mRNA Pairs with Inverse Correlations	Number of miRNA–mRNA Pairs with Inverse Correlations and Opposite Fold Change Between Tumor and Normal Samples
BLCA	998	578
*BRCA*	*20*,*661*	*10*,*101*
*COAD*	*82*	*55*
*ESCA*	*344*	*155*
HNSC	3066	1601
KICH	1039	442
*KIRC*	*10*,*749*	*6189*
KIRP	6143	3190
LIHC	1426	659
*LUAD*	*26*,*380*	*12*,*874*
LUSC	265	171
*PRAD*	*6972*	*3801*
*STAD*	*12*,*892*	*5108*
THCA	1326	744
UCEC	408	214
Total	92,751	45,882

Notes: The italic rows are cancer types included for the analysis in this study in addition to cancer types selected for the analysis in our previous study (Bai et al., 2016).

**Table 2:** The detected “communities” or “clusters” of significant pairs selected for each of 15 selected cancers from TCGA.

Cancer Types	Total Number of Detected Clusters	Number of Detected Significant Clusters (FDR < 0.1)
BLCA	28	2
BRCA	33	8
COAD	20	0
ESCA	42	0
HNSC	96	4
KICH	64	1
KIRC	51	8
KIRP	62	4
LIHC	114	1
LUAD	21	9
LUSC	39	2
PRAD	52	3
STAD	39	8
THCA	57	4
UCEC	70	0
Total	788	54

**Table 3:** The list of matched miRNAs upregulated in human with nonalcoholic fatty liver disease (NAFLD) and their targeted genes.

Gene	miRNA
*ITPKB*	*hsa-mir-106b*
*CD69*	*hsa-mir-106b*
*EPHA4*	*hsa-mir-106b*
*APOBEC3H*	*hsa-mir-106b*
*CYP2U1*	*hsa-mir-106b*
*ZNFX1*	*hsa-mir-106b*
*CNTNAP1*	*hsa-mir-505*
*EFCAB1*	*hsa-mir-505*
*BTG1*	*hsa-mir-505*
*HPRT1*	*hsa-mir-505*
*PAM*	*hsa-mir-505*
*IRF2BP2*	*hsa-mir-505*
*FST*	*hsa-mir-505*
*CLDN23*	*hsa-mir-505*
*SIN3A*	*hsa-mir-20b*
*XPR1*	*hsa-mir-2355*
*C7orf49*	*hsa-mir-2355*
*ZDHHC23*	*hsa-mir-2355*
*VANGL1*	*hsa-mir-2355*
*SSX2IP*	*hsa-mir-584*
*DYNLT3*	*hsa-mir-584*
*ESR1*	*hsa-mir-584*
*ARL15*	*hsa-mir-877*
*MEST*	*hsa-mir-181d*
*TBCC*	*hsa-mir-374b*
*GUCY1A2*	*hsa-mir-551b*
*SCO1*	*hsa-mir-200b*
*CASC4*	*hsa-mir-200b*
*FAM169A*	*hsa-mir-200b*
*UGGT1*	*hsa-let-7b*
*PLEKHA6*	*hsa-let-7b*
*ATP6V1C1*	*hsa-let-7b*

**Table 4:** A list of identified miRNAs and their targeted gene pairs.

Gene	miRNA
*DTNA*	*mir-122*
*SMYD2*	*mir-122*
*IGF2*	*mir-122*
*KYNU*	*mir-122*
*DBNDD1*	*mir-122*
*SYNCRIP*	*let-7c*
*KIF5B*	*let-7c*
*MGAT4A*	*let-7c*
*PDLIM2*	*let-7c*
*LDHD*	*let-7c*
*PLCB1*	*let-7c*
*BDH1*	*let-7c*
*STXBP4*	*let-7c*
*UGGT1*	*let-7b*
*PLEKHA6*	*let-7b*
*ATP6V1C1*	*let-7b*
*CBX7*	*mir-192*
*ZC3H10*	*mir-192*
*RAB2A*	*mir-192*
*TRIM66*	*mir-192*
*MYO1E*	*mir-192*
*ING5*	*mir-192*
*SYAP1*	*mir-192*
*P2RX4*	*mir-29a*
*ZNF286B*	*mir-29a*
*CNDP2*	*mir-29a*
*GPR146*	*mir-29a*
*BMF*	*mir-29a*
*SSTR2*	*mir-29a*
*NLN*	*mir-29a*
*AMICA1*	*mir-29a*
*SYNM*	*mir-29a*
*PRPF3*	*mir-29a*
*CHST10*	*mir-29a*
*ZNF160*	*mir-29a*
*NDN*	*mir-29a*
*MTMR2*	*mir-29a*
*ZNF431*	*mir-29a*
*NAP1L1*	*mir-29a*
*ATP6V0E2*	*mir-29a*
*ATPAF1*	*mir-29a*
*MORF4L1*	*mir-29a*
*PRR3*	*mir-29a*
*CPT2*	*mir-29a*
*DNAJA3*	*mir-29a*
*RIT1*	*mir-29a*
*UCP3*	*mir-29a*
*ZNF35*	*mir-21*
*WDR72*	*mir-21*
*KIAA1804*	*mir-21*
*LAMP2*	*mir-21*
*PFN2*	*mir-21*
*NFASC*	*mir-21*
*FABP4*	*mir-21*
*C7*	*mir-21*
*STK3*	*mir-21*
*RASGRF1*	*mir-132*
*STK3*	*mir-132*
*PFN2*	*mir-132*
*MEST*	*mir-132*
*NCALD*	*mir-132*
*C9orf156*	*mir-132*
*LAMP2*	*mir-99a*
*RCBTB1*	*mir-99a*
*KPTN*	*mir-99a*
*RPS20*	*mir-99a*
*ZDHHC18*	*mir-99a*
*ABCB4*	*mir-200c*
*PGAM1*	*mir-200c*
*SCO1*	*mir-200c*
*IGFBP2*	*mir-145*
*PRPF38A*	*mir-145*
*CDK5RAP3*	*mir-145*
*RBMX*	*mir-145*
*MGLL*	*mir-145*

**Table 5:** A list of miRNAs targeted six genes involved in phospholipase D signaling pathway.

Gene	miRNA
*DGKQ*	*mir-140*
*LPAR2*	*mir-140*
*PDGFRB*	*mir-186*
*PIK3R3*	*mir-151*
*PIK3R3*	*mir-148b*
*PIK3R3*	*mir-589*
*PTGFR*	*mir-107*
*RAPGEF3*	*mir-454*
*RAPGEF3*	*mir-93*
*RAPGEF3*	*mir-25*
*RAPGEF3*	*mir-186*
*RAPGEF3*	*mir-942*

## Supplementary Materials

The following are available online at https://www.mdpi.com/2073-4425/10/9/702/s1, Supplementary file 1: The sequencing data information for TCGA 33 cancer types, Supplementary file 2: Selected anti-correlated miRNA-mRNA pairs passing cutoff criterion (*q* < 0.1) of statistical significance, Supplementary file 3: Clustering results for 15 selected cancers, Supplementary file 4: BLCA cluster similarity result, Supplementary file 5: BRCA cluster similarity result, Supplementary file 6: COAD cluster similarity result, Supplementary file 7: ESCA cluster similarity result, Supplementary file 8: HNSC cluster similarity result, Supplementary file 9: KICH cluster similarity result, Supplementary file 10: KIRC cluster similarity result, Supplementary file 11: KIRP cluster similarity result, Supplementary file 12: LIHC cluster similarity result, Supplementary file 13: LUAD cluster similarity result, Supplementary file 14: LUSC cluster similarity result, Supplementary file 15: PRAD cluster similarity result, Supplementary file 16: STAD cluster similarity result, Supplementary file 17: THCA cluster similarity result, Supplementary file 18: UCEC cluster similarity result, Supplementary file 19: Cluster comparison with three metrices (number_of_neighbors, similarity_percentage, common_pair_percentage).

## Abbreviations

TCGA	The Cancer Genome Atlas
LIHC	Liver hepatocellular carcinoma
KIRC	Kidney renal clear cell carcinoma
KIRP	Kidney renal papillary cell carcinoma
LUAD	Lung adenocarcinoma
BLCA	Bladder urothelial carcinoma
BRCA	Breast invasive carcinoma
COAD	Colon adenocarcinoma
ESCA	Esophageal carcinoma
HNSC	Head and neck squamous cell carcinoma
KICH	Kidney chromophobe
LUSC	Lung squamous cell carcinoma
PRAD	Prostate adenocarcinoma
STAD	Stomach adenocarcinoma
THCA	Thyroid carcinoma
UCEC	Uterine corpus endometrial carcinoma
